# Distributions of Visual Receptive Fields from Retinotopic to Craniotopic Coordinates in the Lateral Intraparietal Area and Frontal Eye Fields of the Macaque

**DOI:** 10.1007/s12264-023-01097-8

**Published:** 2023-08-13

**Authors:** Lin Yang, Min Jin, Cong Zhang, Ning Qian, Mingsha Zhang

**Affiliations:** 1 Key Laboratory of Cognitive Neuroscience and Learning, Division of Psychology, Beijing Normal University, Beijing, 100875 China; 2 Institute of Neuroscience, Key Laboratory of Primate Neurobiology, CAS Center for Excellence in Brain Science and Intelligence Technology, Chinese Academy of Sciences, Shanghai, 200031 China; 3 Department of Neuroscience and Zuckerman Institute, Columbia University, New York, 10027 USA

**Keywords:** Reference frames, Eye-centered, Head-centered, Visual stability

## Abstract

Even though retinal images of objects change their locations following each eye movement, we perceive a stable and continuous world. One possible mechanism by which the brain achieves such visual stability is to construct a craniotopic coordinate by integrating retinal and extraretinal information. There have been several proposals on how this may be done, including eye-position modulation (gain fields) of retinotopic receptive fields (RFs) and craniotopic RFs. In the present study, we investigated coordinate systems used by RFs in the lateral intraparietal (LIP) cortex and frontal eye fields (FEF) and compared the two areas. We mapped the two-dimensional RFs of neurons in detail under two eye fixations and analyzed how the RF of a given neuron changes with eye position to determine its coordinate representation. The same recording and analysis procedures were applied to the two brain areas. We found that, in both areas, RFs were distributed from retinotopic to craniotopic representations. There was no significant difference between the distributions in the LIP and FEF. Only a small fraction of neurons was fully craniotopic, whereas most neurons were between the retinotopic and craniotopic representations. The distributions were strongly biased toward the retinotopic side but with significant craniotopic shifts. These results suggest that there is only weak evidence for craniotopic RFs in the LIP and FEF, and that transformation from retinotopic to craniotopic coordinates in these areas must rely on other factors such as gain fields.

## Introduction

Vision is among the most important sensory modalities by which primates explore the world. Because the fovea, the retinal area with the highest spatial acuity, only covers <5° of visual space, we make frequent saccadic eye movements to bring different parts of the world to the fovea for detailed processing. However, each eye movement changes the projected retinal locations of objects. If the brain only used the retinal information for visual perception, the world would appear to jump around with saccades. As realized by Helmholtz [1], the brain must combine retinal and extraretinal information to achieve visual stability. Two main mechanisms for realizing trans-saccadic visual stability have been proposed. The first is to transform a retinotopic representation into a craniotopic representation with the help of eye-position information [2, 3]. The second is to use corollary discharge of saccade commands to predictively update a retinotopic representation across saccades and link the pre- and post-saccadic images of objects [4]. The first mechanism can be further divided into two possibilities: A craniotopic representation can be realized either implicitly *via* eye-position modulation (gain fields) of retinotopic receptive fields (RFs) [5], or explicitly *via* craniotopic RFs built from retinotopic RFs and eye-position information [6, 7]. In the former case, although RFs remain retinotopic, their gain fields allow them to form an implicit craniotopic representation that can be made explicit through a simple, linear decoder. In the latter case, craniotopic RFs, which are invariant to eye movements by definition, directly provide an explicit craniotopic representation.

Neurons with craniotopic RFs have been reported in the parieto-occipital sulcus (PO or V6) [8], ventral intraparietal area (VIP) [6, 9], and premotor cortex [10]. In particular, Duhamel and colleagues found that the RFs of VIP multi-modality (vision, touch, and ocular proprioception) neurons are distributed continuously from retinotopic to craniotopic coordinates [6, 9], and this distribution can be explained by a recurrent basis-function model [6]. They proposed that the continuous distribution reflects the transformation from retinotopic to craniotopic coordinates. Moreover, based on the model and sensory properties of the lateral intraparietal (LIP), frontal eye fields (FEF), and superior colliculus (SC), they predicted that neurons in these areas have largely retinotopic visual RFs. However, other studies have found that neurons in the LIP and FEF have RFs distributed between retinotopic and craniotopic coordinates, with significant fractions of craniotopic RFs [11–13].

It has also been reported that the proportions of neurons with retinotopic and craniotopic RFs gradually increase and decrease, respectively, from the LIP to FEF to SC [12]. These authors thus argued that the LIP contributes more than the FEF and SC to the transformation of visual information from retinotopic to craniotopic coordinates. However, the LIP, FEF, and SC data they compared were collected from different animals in different labs, and these differences might have affected the results.

Another limitation of previous studies on coordinate representations in the LIP and FEF is that the authors did not map the visual RFs of neurons but instead, only measured their responses to a fixed set of saccade targets arranged along the horizontal dimension [12]. As such, they did not know where the RF centers were or how the RF centers changed with eye fixation. To determine coordinate representations accurately, one has to measure visual responses from a grid of stimulus positions tailored for individual neurons to map their detailed two-dimensional (2D) RFs under different fixations.

In this study, we focused on the questions of whether there is clear evidence for craniotopic RFs in the LIP and FEF and whether the two areas differ from each other in this regard. We applied the same RF mapping and analysis procedures to the LIP and FEF, and the data from the two areas were obtained from the same animals. We focused on the LIP and FEF for two reasons. First, they are among the most important brain areas for sensorimotor integration and thus for coordinate transformation [2, 4]. Second, as we discussed above, previous studies on coordinate representations in these areas are incomplete, with inconsistent conclusions. We aimed to resolve the inconsistency with 2D RF measurements.

## Materials and Methods

### Animal Preparation

Three adult rhesus monkeys (*Macaca mulatta,* one monkey was bought from the Center of Laboratory Macaque in Huangshan, China, two monkeys were bought from Xishan Zhongke Laboratory Animal Co., Ltd in Suzhou, China) weighing 9–11 kg participated in the present study. In each monkey, we implanted eye coils (Crist Instrument, Hagerstown, USA, sample rate at 2.7 kHz) for monitoring the eye position, a head post for restraining head movements, and two chambers (2-cm diameter) for chronically recording extracellular single-unit activity. Recording chambers in the parietal lobe were centered at 3, 10, and 3.2 mm posterior to the interaural plane and 13, 15, and 15 mm lateral from the middle line in the three monkeys, respectively, in order to cover their lateral intraparietal sulci. Recording chambers in the frontal lobe were centered at 28, 18, and 23.5 mm anterior to the interaural plane and 13, 15, and 23.5 mm lateral from the middle line for the three monkeys, respectively, in order to cover their arcuate sulci. The experimental procedures were reviewed and approved by the Ethics Committee at Beijing Normal University.

### Behavioral Tasks

The experiments were conducted in a dark room. Experimental procedures were controlled by the REX (National Institutes of Health, Bethesda, USA) under the QNX (Quick Unix) operating system. All the stimuli were presented on a 55-inch LED monitor (Samsung, Suwon, Korea) with a refresh rate of 60 Hz. Three monkeys (Ba, Mi, and Vd) participated in this study. They sat in monkey chairs facing the screen at a distance of 57 cm so that the screen covered a 90° (horizontal) × 60° (vertical) area of the visual field. The eye positions were monitored by the scleral search coil technique (Crist Instrument Sclera) during the behavior tasks. Before an experiment, the eye positions were calibrated by requiring monkeys to sequentially look at nine fixation points.

**Fig. 1:**
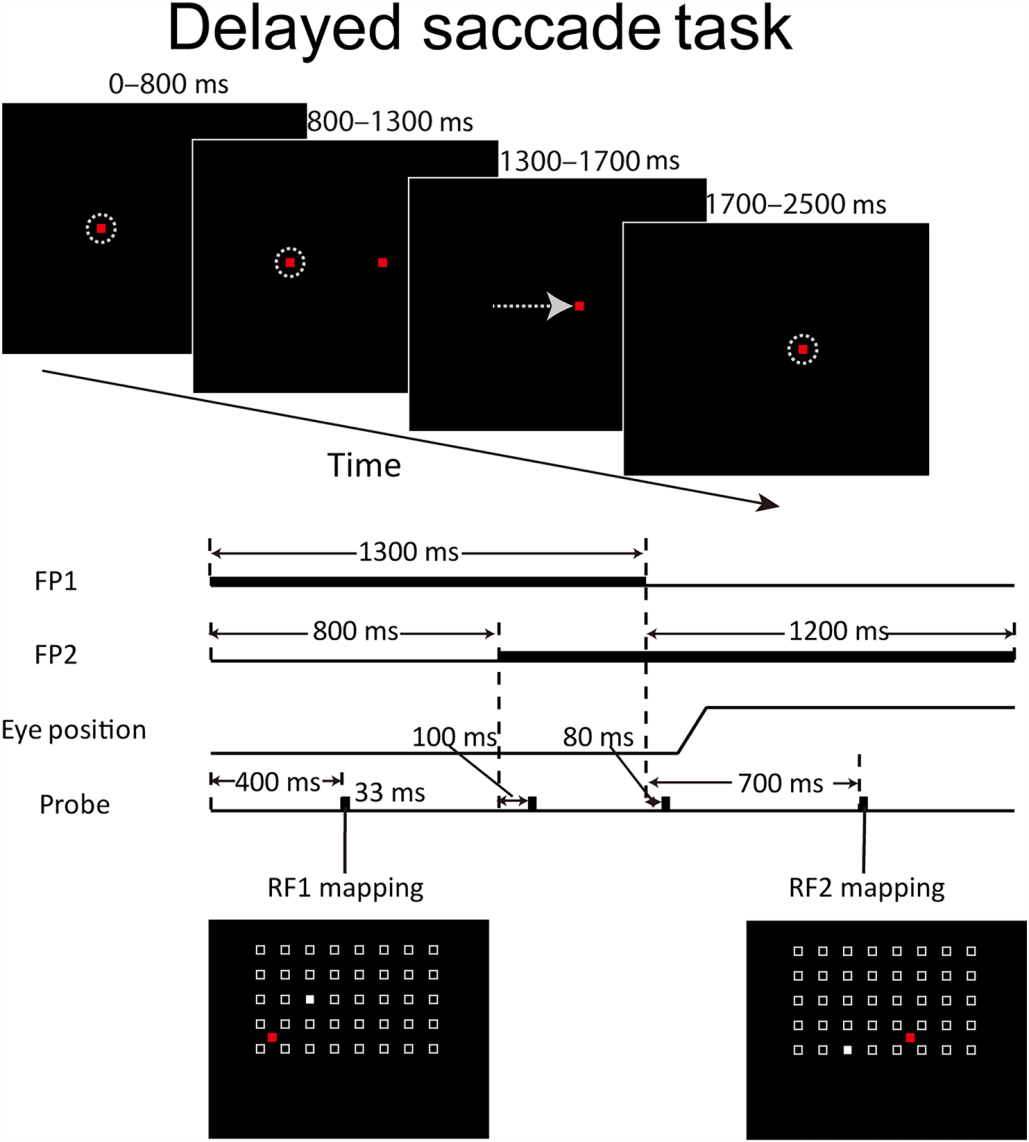
The delayed saccade task. Upper panel: the temporal sequence of the task. The two red squares represent the first and second fixation points (FP1 and FP2). The dashed circles denote monkeys’ eye positions in each time period. The arrow represents a saccade from FP1 to FP2. Lower panel: the detailed timing of the events in the task. The four rows represent the time courses of FP1, FP2, the eye position, and the probes. The location of the visual probe (filled white square) was randomly chosen from a matrix (open squares).

The experimental paradigm was designed for a different purpose but since we mapped neurons’ RFs at two different fixation points, we used this aspect of the data for the current study. After isolating a single unit with a template-matching method, we did a pilot mapping of its RF. If the neuron showed a clear RF, we then used a delayed saccade task (Fig. 1) to map its RFs at two fixation points. A trial began with a red square (0.3° × 0.3°) presented on the screen as the first fixation point (FP1). Monkeys were required to look at the FP1 within 1000 ms; 800 ms after the fixation, another red square (0.3° × 0.3°) was presented at a different location on the screen serving as the second fixation point (FP2). The monkeys were required to keep fixation at FP1 until its disappearance (1300 ms after acquiring the fixation), then to make a saccade to FP2 within 400 ms, and to keep fixation at FP2 until its disappearance (1200 ms after FP1 disappearance). Correct trials were rewarded with juice drops. A trial was aborted if any of the above requirements on the eye position was violated. Four visual probes (white squares, each 1° × 1° in size and 33 ms in duration) were sequentially presented at four locations randomly chosen from a probe grid on the screen. The size and location of the 2D grid for sampling responses were tailored according to the size and location of a given neuron’s RF. The grid size varied from 4 × 5 to 10 × 11 positions with 5 × 8 being the most common. The distance between two adjacent locations was 6° along both horizontal and vertical axes. They were presented at different epochs of the trial as follows: 500 ms after fixating at FP1, 100 ms after FP2 onset, 80 ms after FP1 offset, and 700 ms after FP1 offset. In the present study, only the responses to the first and fourth probes were used to calculate the neuron’s RFs at FP1 and FP2. These probes were well separated from the saccade in time (Fig. 1) and the two RFs so measured were thus the RFs for the two fixation points. The responses from the second and third probes were for other purposes and are not included in this paper. For each isolated neuron, we recorded its responses for one session which consisted of an average of 565 trials. FP1 and FP2 were either horizontally or vertically separated. The distance between them ranged from 10° to 30° with 15° being the most common. For a given cell, FP1, FP2, and the stimulus grid were fixed for all trials.

### Recording Procedures

Single-unit activity was recorded using insulated tungsten microelectrodes (0.3–1.0 MΩ, FHC, Bowdoin, USA). Stainless-steel guide tubes were used to protect electrodes when breaking through the dura. Then the electrodes were driven by a micromanipulator (NAN Instruments, Nof Hagalil, Israel) into the cortex. Neuronal activity was amplified and filtered (268–8036 Hz) by AlphaLab SnR (Alpha Omega, Nof Hagalil, Israel).

Before starting to collect neuronal data, we first identified the locations of the LIP and FEF according to their physiological properties and MRI (magnetic resonance imaging) images. We applied a memory-guided saccade task to localize the LIP by finding neurons with persistent activity in the delay period, as reported previously [14]. To localize the FEF, we used sub-threshold electrical micro-stimulation (100 ms, 0.05 mA, biphasic pulses) that evoked saccades with fixed amplitude and direction [15, 16]. We made MRI scans for two of the three monkeys (Mi and Vd) after several recording sessions. The scans confirmed that the recording sites for the LIP and FEF were within the lateral bank of the intraparietal sulcus and the anterior bank of the arcuate sulcus, respectively.

### Data Analysis

#### Eye Positions During Fixation

To ensure that the eyes were relatively still when we measured a neuron’s response to a probe, we calculated offline the eye velocity over a 200-ms window from −50 ms to 150 ms relative to the probe onset. We excluded trials in which the velocity exceeded 30°/s for longer than 20 ms. We then averaged the eye positions within the 200 ms window as the eye position for the response to the probe.

During the experiment, we used a 6° × 6° fixation window for monkeys Mi and Vd, and a 10° × 10° to 14° × 14° window for monkey Ba because the eye coils for monkey Ba were unstable. To ensure accurate fixations, we excluded the trials with eye positions outside a 4° × 4° window centered at the average eye position for all monkeys. Overall, we excluded 6.93%, 1.21%, and 3.07% of trials for monkeys Ba, Mi, and Vd, respectively.

#### The Criterion for Selecting Neurons with Visual Responses

We screened for neurons with clear visual responses as follows. We used the responses in the 100-ms interval prior to the probe onset as the baseline and the responses 50 to 150 ms after probe onset as the response to the probe. If a neuron’s strongest probe responses (across locations) were significantly greater than the baseline (Wilcoxon signed rank test), we considered it as having visual responses. With this procedure, we excluded just one neuron from the FEF and no neurons from the LIP.

#### The Criterion for Defining RF

For a neuron that passed the above screening, we interpolated (bilinearly) its mean responses across probe locations and resampled the responses with a spatial resolution of 1° × 1°. Then, we normalized the responses according to:

1 $$ r_{xy}{\prime} { } = { }\frac{{r_{xy} - r_{{{\text{min}}}} }}{{r_{{{\text{max}} }} - r_{{{\text{min}}}} }} $$

where *x* and *y* denote the probe location under the screen coordinates which were craniotopic for head-fixed animals. $r_{xy}$ and $r_{xy}{\prime}$ denote the original and normalized visual responses for probe location (*x*, *y*). $r_{{{\text{max}}}}$ and $r_{{{\text{min}}}}$ denote the maximum and minimum responses after the interpolation.

We determined the RF as the area where normalized responses were greater than or equal to a threshold of 50%. We excluded neurons for further analysis if the trial number was <4 at any probe location within any of their two RFs. With this procedure, we excluded 66 neurons from the LIP and 69 neurons from the FEF. We also verified our results with a threshold of 75% and 85% for defining RF.

#### The RF Center

We then calculated the center of mass of an RF as the center of the RF. Specifically,

2 $$ \overline{x}{ } = { }\frac{1}{S}\mathop \sum \limits_{x} \mathop \sum \limits_{{\text{y}}} r_{xy}{\prime} x $$

3 $$ \overline{y}{ } = { }\frac{1}{S}\mathop \sum \limits_{x} \mathop \sum \limits_{{\text{y}}} r_{xy}{\prime} y $$

4 $$ S{ } = { }\mathop \sum \limits_{x} \mathop \sum \limits_{{\text{y}}} r_{xy}{\prime} $$

where $\overline{x}$ and $\overline{y}$ are the *x* and *y* positions of the RF center.

#### The Displacement Index Vector

**Fig. 2:**
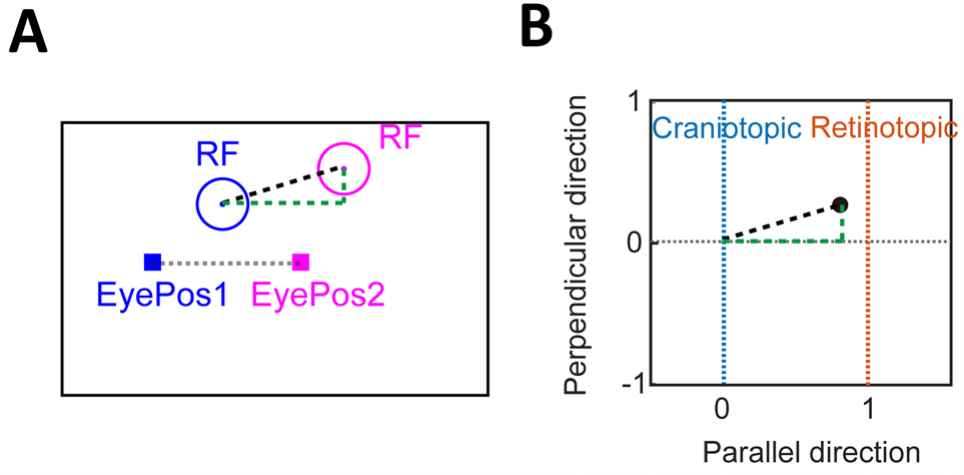
Schematic for the calculation of the displacement index (DI) vector. **A** We first rotated the axis linking the mean eye positions (EyePos1 and EyePos2) for the two fixation points horizontally, and measured the displacement vector (dashed black line) from the RF1 center to the RF2 center. The horizontal and vertical components of the vector are indicated by the dashed green lines. **B** We then divided the displacement vector by the distance between EyePos1 and EyePos2 (the length of the gray line in panel A) to obtain the DI vector (dashed black line). The horizontal and vertical components of the DI vector (dashed green lines) are referred to as* x* index and* y* index in the text. The orange and blue vertical lines indicate the *x*-index values for perfectly retinotopic and craniotopic neurons, respectively.

We measured the RFs of each neuron on the screen while the monkeys fixated at two different fixation points on the screen. The two fixation points were aligned either horizontally or vertically. We re-numbered the two fixation points as FP1 and FP2 from left to right in the former case and from lower to upper in the latter case, and denoted the RFs of a given neuron at the two FPs as RF1 and RF2. For population analysis, we standardized the configurations by rotating the axis linking the mean eye positions at FP1 and FP2 to horizontal. We divided the displacement vector between the two RF centers by the distance between the two mean eye positions (Fig. 2A). The resulting displacement index (DI) vector, with a horizontal (*x*) and a vertical (*y*) component, indicates the coordinate system of the neuron. The* x* and* y* axes are parallel and perpendicular to the saccade directions, respectively. The RF of a perfectly craniotopic neuron would not change with the fixation on the screen. The RF displacement vector would be 0 and the index vector would be (0, 0) (i.e., both its* x* and* y* components would be 0). In contrast, the RF of a perfectly retinotopic neuron would be displaced by the same amount as the displacement of the eye, and the index vector would be (1, 0) (Fig. 2B). This index vector is a 2D version of the index in Avillac *et al*. [6]

#### The Correlation-Based Index

In addition to the DI vector above, we also calculated the following correlation-based index, introduced by Caruso *et al*. [11], as another indicator of a coordinate system:

$$ r{ } = { }\frac{1}{n}\mathop \sum \limits_{x} \mathop \sum \limits_{{\text{y}}} \frac{{\left( {r_{1,xy} - \overline{{r_{1} }} } \right)\left( {r_{2,xy} - \overline{{r_{2} }} } \right)}}{{\left| {r_{1,xy} - \overline{{r_{1} }} } \right|\left| {r_{2,xy } - \overline{{r_{2} }} } \right|}} $$

where $r_{1,xy}$ and $r_{2,xy}$ denote the visual responses at the probe location (*x*, *y*) when the monkeys fixated at FP1 and FP2, respectively*.* $\overline{{r_{1} }}$ and $\overline{{r_{2} }}$ denote the average visual responses across all probe locations when the monkeys fixated at FP1 and FP2, respectively. *n* denotes the number of probe locations that were used for calculating the index. Note that Caruso *et al*. [11] used a single $\overline{r}$ for two fixation points by averaging responses across them. We calculated $\overline{{r_{1} }}$ and $\overline{{r_{2} }}$ for the two fixation points separately because in our case, the response amplitude varied greatly across the fixations, a reflection of eye-position gain fields [2]. The index measures the spatial correlation between RF1 and RF2. It was calculated separately under a craniotopic (screen) coordinate and a retinotopic coordinate (with the two fixation points aligned together) for the probe positions. They are referred to as the craniotopic index and retinotopic index, respectively. For a perfectly craniotopic neuron and a perfectly retinotopic neuron, the craniotopic index and retinotopic index, respectively, would reach the maximum value of one.

For most of the recorded neurons, the probe locations for a pair of RFs were not exactly aligned when the corresponding FPs were aligned to represent the RFs in the retinotopic coordinate. To calculate the correlation, we interpolated the visual responses across probe positions and then resampled the responses (with a 1° × 1° spatial resolution) from the same set of grid locations for the two RFs. Importantly, we used the same number of re-sampled locations for calculating the craniotopic index and retinotopic index to avoid any potential bias.

We used the following bootstrap method to determine a neuron’s coordinate system. Using a neuron’s responses from repeated trials as the probability distribution at each probe location, we re-sampled, with replacement, 1000 sets of visual responses across the probe locations, with the trial number at each probe location equal to the actual trial number at that location, and calculated 1000 craniotopic indices and 1000 retinotopic indices. Then, we calculated the 95% confidence interval (CI) for each index distribution. We compared the actual retinotopic index of each neuron with the 95% CI of re-sampled craniotopic indices, and its actual craniotopic index with the 95% CI of the re-sampled retinotopic indices. We also compared each index distribution with 0. We defined a neuron as craniotopically dominated if its craniotopic index was significantly greater than the retinotopic index and 0. And we defined a neuron as retinotopically dominated when its retinotopic index was significantly larger than its craniotopic index and 0. Otherwise, the neuron was considered intermediate.

## Results

In total, we recorded 346 and 312 neurons from the LIP and FEF, respectively. For each neuron, we measured two RFs (RF1 and RF2) when the monkeys fixated on two different fixation points (FP1 and FP2). After screening for neurons with visual responses and sufficient trial numbers (Methods), we had 280 and 242 neurons in the LIP and FEF, respectively.

### Example Neurons with RFs in Retinotopic, Craniotopic, and Intermediate Coordinates in the LIP and FEF

**Fig. 3:**
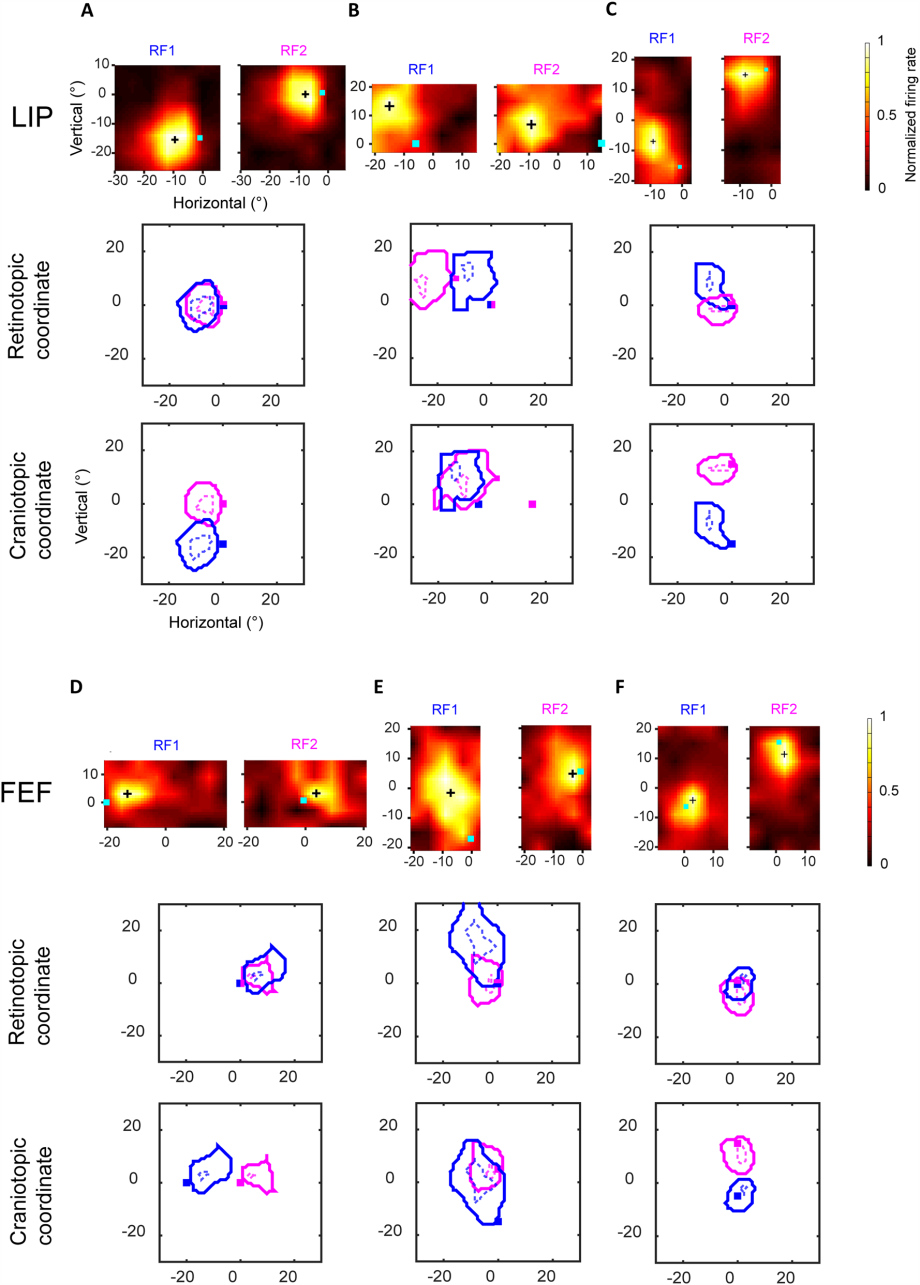
Example LIP and FEF neurons. Three LIP neurons (**A–C**) and three FEF neurons (**D–F**) whose RFs approximately followed retinotopic, craniotopic, and intermediate coordinate representations. In each panel, the first row shows heatmaps of the neuron’s RF1 and RF2. The cyan squares denote the fixation points, and the black crosses represent the RF centers. The second and third rows show the RF contours (solid: 50% of the peak; dotted: 85% of the peak) in retinotopic and craniotopic coordinate systems. The contours for RF1 and RF2 are blue and magenta, respectively.

In Fig. 3, we show three example LIP neurons in panels A–C, and three example FEF neurons in panels D–F. For each brain area, we selected the three neurons that show approximately retinotopic, craniotopic, and intermediate RF representations, respectively. In each panel, the first row shows the heat maps of RF1 and RF2 (the neuron’s RFs for the fixation points FP1 and FP2, respectively), the second row shows the RF1 and RF2 contours at 50% (solid) and 85% (dashed) of the peak normalized response, respectively, in retinotopic coordinates, and the third row shows the same RF1 and RF2 contours in craniotopic coordinates. The first neuron in each area (panels A and D) had well-overlapped RF1 and RF2 in the retinotopic coordinates but not in the craniotopic coordinates. In contrast, the second neuron in each area (panels B and E) had well-overlapped RF1 and RF2 in the craniotopic coordinates but not in the retinotopic coordinates. Finally, the third neuron in each area (panels C and F) showed an intermediate behavior. Therefore, these neurons showed approximately retinotopic, craniotopic, and intermediate RF representations, respectively.

### The RFs of LIP and FEF Neurons Are Distributed from Retinotopic to Craniotopic Representations

**Fig. 4:**
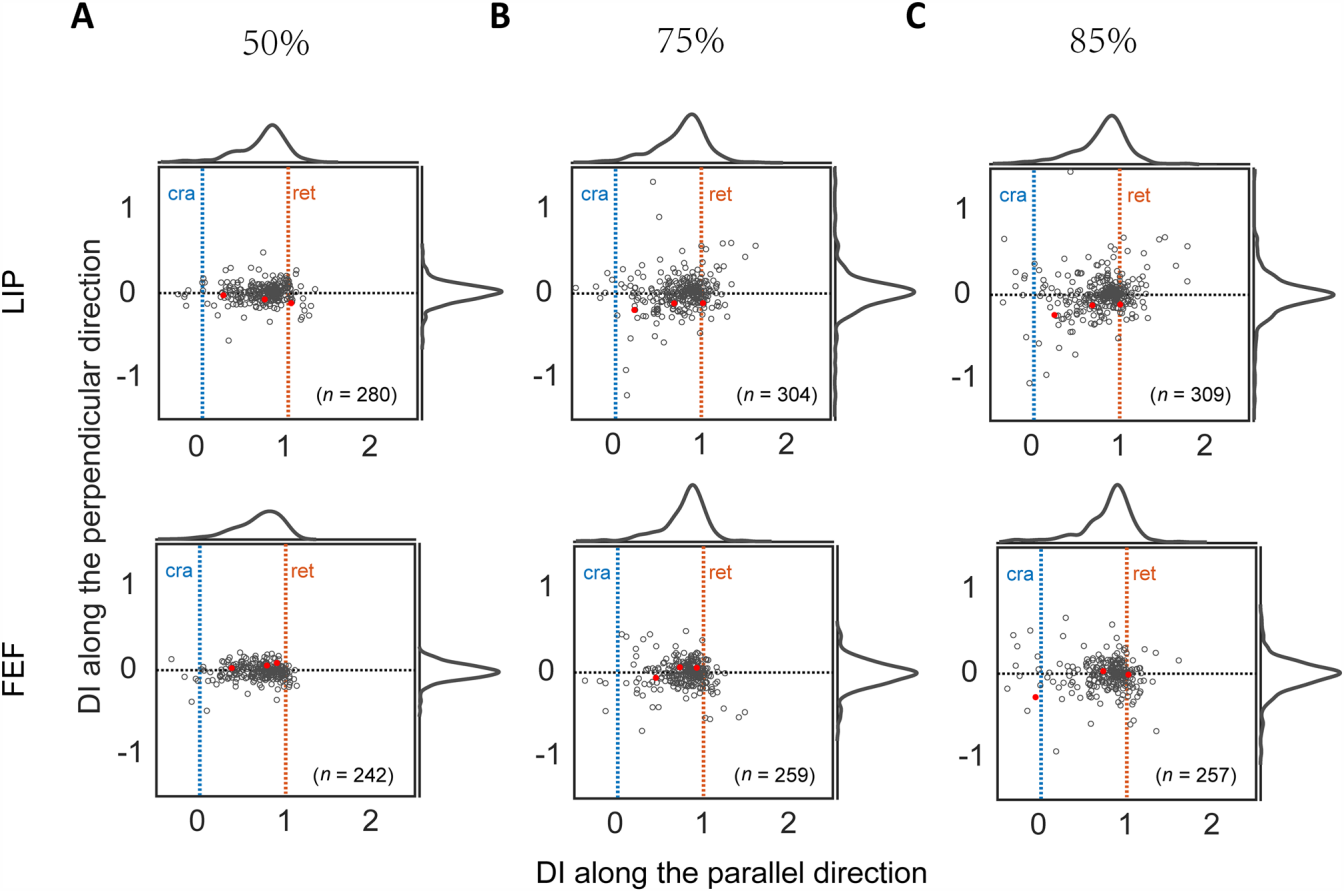
The DI distributions of LIP and FEF neurons. The results for the LIP and FEF are shown in the two rows, and those for the 50% (**A**), 75% (**B**), and 85% (**C**) RF thresholds are shown in the three columns. In each panel, the horizontal and vertical axes represent the* x* and* y* components of the DIs, respectively. The blue and orange vertical lines indicate the *x*-index values for perfectly retinotopic (ret) and craniotopic (cra) RFs, respectively. The red dots indicate example neurons in Fig. 3. The marginal distributions of the* x* and* y* indices are also shown.

We next assessed the population behaviors of LIP and FEF neurons. We first standardized the recording configurations so that for all recorded neurons, the two mean eye positions (for the two FPs) were aligned horizontally. Then for a neuron’s two RFs, we defined a DI vector, with a horizontal (*x* index) and vertical (*y* index) component, to characterize the neuron’s coordinate representation (see Methods). For perfectly craniotopic and retinotopic RF representations, the* x* index should be 0 and 1, respectively, and the* y* index should be 0. Fig. 4 shows the actual DI distributions for LIP (top row) and FEF (bottom row) neurons. The DI calculation depends on the RF region used to calculate the RF center. In Fig. 4, we used the region where responses to probes were above a threshold of 50% of the peak normalized response. To demonstrate the robustness of our findings, we repeated the calculation but with the response threshold changed to 75% (Fig. 4B) and 85% (Fig. 4C). For all six panels, the* x* indices were distributed from 0 to 1 with clusters around 1, indicating coordinate representations from craniotopic to retinotopic, with a strong bias toward the retinotopic side. However, in all six panels, the mean* x* indices were significantly different from 1 (the mean* x* indices in LIP and FEF were: 0.68 and 0.65 at the threshold of 50%, 0.74 and 0.73 at the threshold of 75%, and 0.74 and 0.73 at the threshold of 85%, Wilcoxon signed rank test, *P* <0.01 for all 6 panels), indicating that the RF representations in the LIP and FEF are not completely retinotopic. In contrast, the* y* indices in all six panels were distributed around 0. For the LIP, the mean* y* indices were not significantly different from 0 (the mean* y* indices were −0.01 at the three thresholds, Wilcoxon signed rank test, *P* >0.05 for three panels). The mean* y* indices in the FEF were significantly different from 0 (the mean* y* indices were −0.03 at three thresholds, Wilcoxon signed rank test, *P* <0.01 for three panels in the FEF). The variance of* y* indices were smaller than that of* x* indices (two-sample *F*-test, *P* <0.01 for all six panels). We conclude that the evidence for fully craniotopic RFs in the LIP and FEF is weak, and that RFs in these areas are mostly in retinotopic coordinates but with a significant shift in the craniotopic direction.

**Fig. 5:**
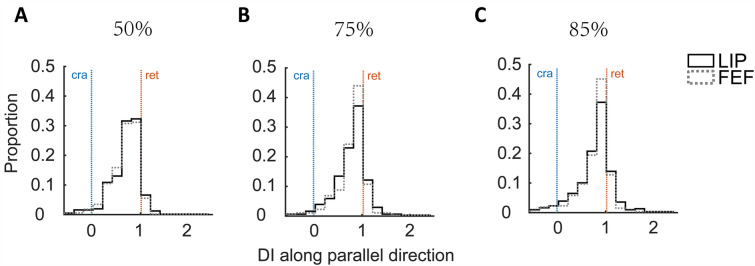
Distributions of the* x* component of the DI. **A–C** The three panels show the results for the three RF thresholds (50%, 75%, and 85%). In each panel, the LIP and FEF distributions are shown as solid and dashed histograms, respectively. The blue and orange vertical lines indicate the *x*-index values for perfectly craniotopic (cra) and retinotopic (ret) RFs, respectively.

We next compared the* x* component of the DI distributions between LIP and FEF (Fig. 5). The differences between the means (Wilcoxon rank-sum test, *P* >0.05 for all three thresholds) and between the distributions (Kolmogorov–Smirnov test, *P* >0.05 for all three thresholds) were not significant.

**Fig. 6:**
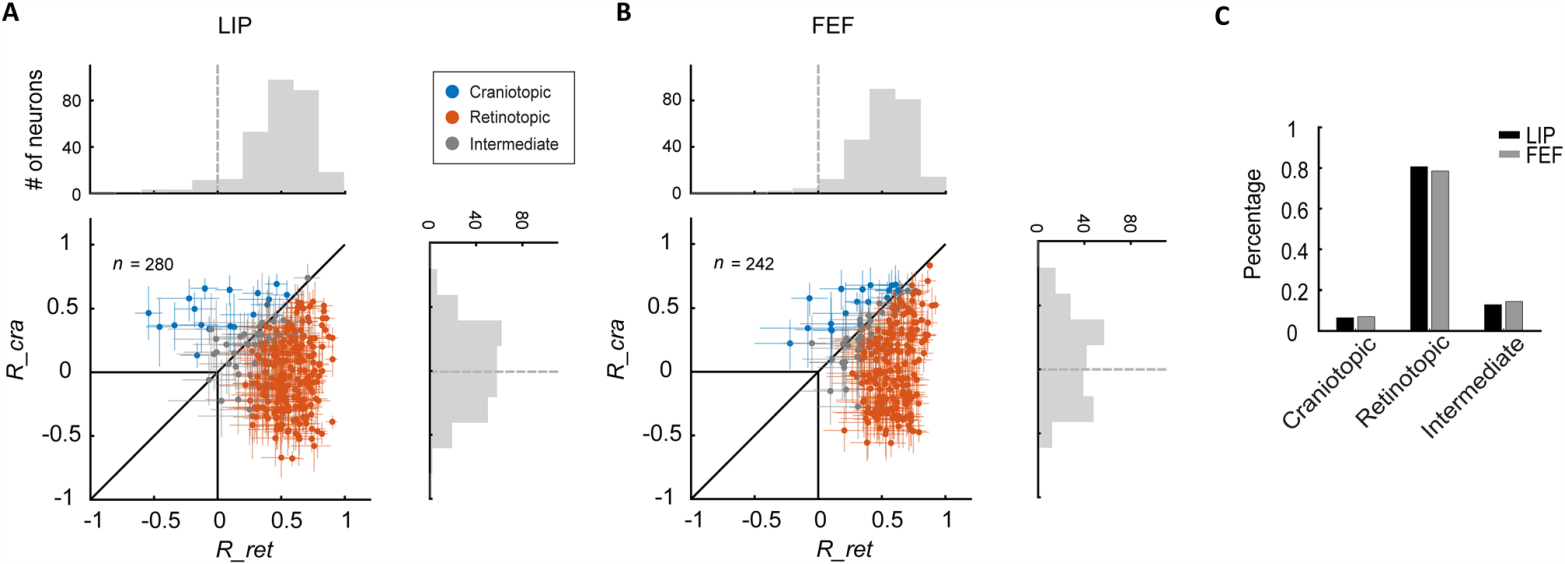
The distributions of retinotopic and craniotopic correlation indices in the LIP and FEF. For each neuron in the LIP (**A**) and FEF (**B**), its craniotopic correlation index (*R*_cra) is plotted against its retinotopic correlation index (*R*_ret). The dots and the error bars indicate the mean and 95% confidence intervals. The marginal distributions are also shown. The orange, blue, and gray dots represent retinotopically dominated neurons, craniotopically dominated neurons, and intermediate neurons, respectively. **C** The proportions of the three types of neurons in the LIP (black) and FEF (grey).

In addition to the DI, we also applied the craniotopic and retinotopic correlation indices of Caruso *et al*. [11, 12] (*R_*cra and *R_*ret) to measure RF coordinate representations (see Methods). *R_*cra and *R_*ret reach the maximum value of 1 for perfectly craniotopic and retinotopic neurons, respectively. In Fig. 6, *R_*cra is plotted against *R_*ret for neurons in the LIP (panel A) and FEF (panel B), together with the marginal distributions of the indices. In both areas, the mean retinotopic correlation index was significantly larger than the mean craniotopic index (Wilcoxon signed rank test, *P* <0.01 for both LIP and FEF). We then classified the neurons into craniotopic, retinotopic, and intermediate categories according to their correlation indices (see Methods). The result (Fig. 6C) showed that most neurons in both areas are retinotopic, consistent with our analysis above with the DI (Fig. 4).

We also compared the proportions of craniotopic, retinotopic, and intermediate neurons (classified according to the correlation indices) between LIP and FEF. Again, the difference between the two areas was not significant (Fig. 6C; *χ*^2^ test, *P* = 0.99).

## Discussion

In this study, we measured the 2D visual RFs of single neurons in the LIP and FEF while the monkeys were fixating at two different locations on the screen. By comparing the RFs measured from the different fixations, we found that the LIP and FEF neurons distributed from retinotopic to craniotopic representations of their RFs. The distributions were dominated by the retinotopic representation, but with a significant shift in the craniotopic direction. In addition, the distributions were not significantly different between LIP and FEF.

### Comparison with Previous Studies on LIP and FEF Coordinate Representations

We found predominantly retinotopic RF representations in both LIP and FEF, consistent with the predictions by Avillac *et al*. [6]. However, Caruso *et al*. [11, 12] reported larger proportions of craniotopic and intermediate neurons and a smaller proportion of retinotopic neurons, compared with our results (Fig. 6C). This difference might be attributable to the different task designs used in our and their studies. First, as noted in the Introduction, we mapped neurons’ 2D RFs in detail while they did not. Second, the probe stimuli for mapping RFs in our experiments were task-irrelevant (Fig. 1), whereas in Caruso *et al*.’s study [12], monkeys were required to make a saccade to the probe after the disappearance of the initial fixation point. Therefore, the monkeys in our and their studies likely paid less and more attention, respectively, to the probe stimuli. It has been reported that human visual areas (the middle temporal, medial superior temporal, lateral occipital, and V6 area) show stronger retinotopic selectivity (and weaker craniotopic selectivity) when subjects pay less attention to the stimuli [17]. Similarly, Chen *et al*. [18, 19] found a larger proportion of retinotopic RFs in the VIP than did Avillac *et al*. [6] and Duhamel *et al*. [9], and suggested lower and higher levels of attention to the stimuli in the two sets of studies, respectively, might be responsible for the different results. Thus, the difference between our study and that of Caruso *et al*. [11, 12] in the LIP and FEF might also result from the different levels of attention to the stimuli. Another related study is that of Yang and Gu [20] who recorded neurons in the FEFsem (frontal eye field, smooth eye movement subregion) and MSTd (dorsal medial superior temporal area) when monkeys passively viewed optic-flow stimuli that contained heading direction information. They found that neurons’ heading direction tuning became more craniotopic after the monkeys were trained to estimate the heading direction of optic flow.

Third, we mapped RFs with visual probes only whereas Caruso *et al*. [12] randomly interleaved trials with visual and auditory probes. Since auditory stimuli are encoded in a craniotopic reference frame, they might provide a context to enhance the craniotopic representation of visual stimuli according to the multi-sensory integration model of Avillac *et al*. [6].

Caruso *et al*. [11, 12] also reported a larger fraction of craniotopic RFs in the LIP than in the FEF. However, we did not find a significant difference between the coordinate representations in the two areas. Further investigations are needed to determine factors responsible for the discrepancy between the two studies.

LIP and FEF neurons can be classified into visual, motor, and visuomotor, depending on whether or not they have visual responses, saccade-related responses, or both. Since the visuomotor neurons must be involved in sensorimotor integration, it would be interesting to investigate their RF coordinate representation separately. Unfortunately, our study cannot address the issue because we recorded cells with only a single saccade direction and did not use a full range of saccade directions to map the motor fields for the classification of the cells. A previous study did investigate the coordinate representations of visual and visuomotor neurons separately and found similar patterns [12]. However, since the study did not measure either the visual RFs or the motor fields of the neurons completely (e.g., no downward saccade directions were included), further studies are needed to fully resolve the issue.

We used two indices to quantify the coordinate representations of neuronal RFs: displacement index and correlation index. The calculation of the DI vector is relatively simple: For a neuron’s pair of RFs recorded with any two fixation points, in the screen coordinate, one first determines the displacement vector between the two RF centers, and then divides the vector by the distance between the two fixations. After standardizing the recording configuration so that the fixation points are horizontally separated, we can then use the horizontal component of the DI to measure a neuron’s coordinate representation. In contrast, we noted that the calculation of the correlation index can be easily biased if not done properly. Again, consider a neuron’s pair of RFs recorded with two (horizontally separated) fixation points, in the screen coordinates. One can then calculate the correlation coefficient between the RFs under various horizontal shifts between the two RFs. When the shift equals zero, the correlation coefficient represents the craniotopic index. When the shift equals the distance between the fixations, the correlation coefficient represents the retinotopic index (as defined in Methods). However, the values of these correlation indices are sensitive to how the measured responses are re-sampled after spatial interpolation. If, for example, one samples both RFs from the same screen (craniotopic) region, then the calculation of the craniotopic index does not need zero padding whereas the calculation of the retinotopic index does. Since the background activities of both RFs are below the corresponding mean responses, their point-by-point products are positive and thus contribute more to the craniotopic index than to the retinotopic index, making the neuron appear artificially more craniotopic. If, instead, one samples both RFs from the same retinotopic region, then the calculation of the retinotopic index does not need zero padding whereas the calculation of the craniotopic index does, making the neuron appear artificially more retinotopic. We avoided this problem by sampling from a large enough region and then using subsets of exactly the same number of sampled points, without zero padding, to calculate both craniotopic and retinotopic indices [11, 12]. Another problem may occur when a single mean response of a neuron’s RFs is used. If the two RFs differ greatly in their response levels (because of eye-position gain modulation, for example), then one RF has many points below the mean and the other RF has many points above the mean, leading to an artificially more negative correlation coefficient. We avoided this problem by calculating a separate mean response for each RF.

### Mechanisms of Coordinate Transformation

As we noted in Introduction, there have been two proposals for transforming a retinotopic coordinate into a craniotopic coordinate. A craniotopic representation can be realized either implicitly *via* eye-position modulation (gain fields) of retinotopic RFs [5], or explicitly *via* craniotopic RFs [6, 7]. Zipser and Andersen [5] trained feedforward neural networks whose hidden layer takes retinotopic visual representation and eye position information as inputs and produces craniotopic visual representations as outputs. They found that hidden units in the trained networks have eye-position gain fields similar to those found in parietal neurons. They argued that the explicitly craniotopic output units do not necessarily have a counterpart in the brain and that the gain-field modulated hidden units, like parietal neurons, form an implicit craniotopic representation that could directly drive motor systems. Avillac *et al*. [6] also combined retinotopic visual representation and eye-position information to produce a craniotopic visual representation in their model. However, they viewed the craniotopic output units as the counterpart of VIP neurons with craniotopic RFs. Moreover, in their model, the craniotopic output units send feedback inputs to the hidden units. The relative strengths of the retinotopic and craniotopic inputs to the hidden units can be adjusted to produce various intermediate RF shifts between the two coordinate systems. Our findings of largely retinotopic RFs in the LIP and FEF suggest that these areas must rely on eye-position gain fields to achieve an implicit craniotopic representation [2]. On the other hand, we also found that LIP and FEF neurons showed significant RF shifts in the craniotopic direction, and these shifts might be produced by feedback from craniotopic RFs in the VIP [6, 9]. However, if gain fields in the LIP and perhaps other areas already produce fully craniotopic RFs in the VIP, what is the function of the VIP feedback to produce only slightly craniotopic RFs in the LIP? Further studies are needed to resolve this puzzle.
