# Correction: Distributions of Visual Receptive Fields from Retinotopic to Craniotopic Coordinates in the Lateral Intraparietal Area and Frontal Eye Fields of the Macaque

**DOI:** 10.1007/s12264-024-01176-4

**Published:** 2024-02-06

**Authors:** Lin Yang, Min Jin, Cong Zhang, Ning Qian, Mingsha Zhang

**Affiliations:** 1 Key Laboratory of Cognitive Neuroscience and Learning, Division of Psychology, Beijing Normal University, Beijing, 100875 China; 2 Institute of Neuroscience, Key Laboratory of Primate Neurobiology, CAS Center for Excellence in Brain Science and Intelligence Technology, Chinese Academy of Sciences, Shanghai, 200031 China; 3 Department of Neuroscience and Zuckerman Institute, Columbia University, New York, 10027 USA

**Correction to: Neurosci. Bull.** 10.1007/s12264-023-01097-8

In the Original Publication, the article title was incorrectly given as 'Distributions of Visual Receptive Fields from Retinotopic to Craniotopic Coordinates in the lateral intraparietal and frontal eye field of the Macaque' but should have been 'Distributions of Visual Receptive Fields from Retinotopic to Craniotopic Coordinates in the Lateral Intraparietal Area and Frontal Eye Fields of the Macaque'.

"The original article has been corrected.".

